# Loss of Sidt2 in Mice Resembles Danon Disease and Results in Induction of a Type I Interferon Response with Increased Apoptosis

**DOI:** 10.3390/ijms27209158

**Published:** 2026-10-15

**Authors:** Marilou H. Barrios, Tan A. Nguyen, Peter J. Houweling, Daniel Frank, Fleur C. Garton, Hart G. Lidov, Ellen Tsui, Alexandra L. Garnham, Amrut V. Ambardekar, Gordon K. Smyth, Luisa Mestroni, Joseph C. Cleveland, Matthew R. G. Taylor, Kathryn N. North, Craig P. Hunter, Ken C. Pang

**Affiliations:** 1 The Walter and Eliza Hall Institute of Medical Research, Parkville, VIC 3050, Australia; 2 Department of Medical Biology, University of Melbourne, Parkville, VIC 3050, Australia; 3 Murdoch Children’s Research Institute, Parkville, VIC 3052, Australia; 4 Department of Paediatrics, University of Melbourne, Parkville, VIC 3052, Australia; 5 Institute for Molecular Bioscience, The University of Queensland, Brisbane, QLD 4072, Australia; 6 Department of Pathology, Boston Children’s Hospital, Boston, MA 02115, USA; 7 Department of Medicine, Division of Cardiology, University of Colorado Anschutz Medical Campus, Aurora, CO 80045, USA; 8 Department of Surgery, Division of Cardiovascular Surgery, University of Colorado Anschutz Medical Campus, Aurora, CO 80045, USA; 9 Department of Molecular and Cellular Biology, Harvard University, Cambridge, MA 02138, USA; 10 Royal Children’s Hospital, Parkville, VIC 3052, Australia

**Keywords:** Sidt2, Danon disease, RNAutophagy, muscle, apoptosis, dsRNA, linc-MD1, interferon response

## Abstract

SIDT2 is a lysosomal membrane protein that—together with LAMP2—has been implicated in a recently described process known as RNAutophagy. Mutations in LAMP2 have long been known to cause Danon disease, a rare lysosomal storage disorder that affects multiple organ systems including brain and skeletal muscle. Recently, we have shown that loss of SIDT2 function in both humans and mice causes brain dysfunction, and it has been previously observed that loss of SIDT2 in mice also leads to muscle damage and reduced muscle strength. Here, we show that changes in the skeletal muscle of Sidt2^−/−^ mice are reminiscent of those seen in Danon disease as evidenced by accumulation of autophagic vacuoles with sarcolemmal features, selective atrophy of fast twitch fibers, and a shared alteration in gene expression. Moreover, we find that loss of SIDT2 not only leads to impaired autophagy in skeletal muscle as previously described but also stimulates a type I interferon response and increased apoptosis.

## 1. Introduction

Lysosomal storage disorders (LSDs) are a heterogeneous group of >70 conditions, which arise due to genetic defects that impair normal lysosomal degradation within the cell [1]. The clinical features of LSDs vary widely but frequently include intellectual disability, neurodegeneration, muscle weakness and hepatomegaly [2,3], reflecting the numerous organs that can be affected, such as the brain, skeletal muscle and liver.

SIDT2 (SID1 Transmembrane Family Member 2) is an 832-amino-acid, multi-pass transmembrane protein that is well conserved across mammalian species and localizes to lysosomes [4]. Previous analyses indicate that SIDT2 contains multiple YxxΦ motifs, which interact with adaptor protein complexes and are crucial for its lysosomal localization [5]. Although SIDT2 has not previously been implicated in the etiology of LSDs, accumulating evidence from both humans and mice is consistent with SIDT2 being important for normal lysosomal degradation within the cell.

For example, we recently reported a case involving a child with biallelic loss of SIDT2 function and progressive neurological disease, characterized by cerebellar ataxia, dystonia, and cognitive decline [6]. Fibroblasts from the child demonstrated lysosomal dysfunction, including the accumulation of markers such as p62 and LC3B. These cellular changes were also observed in the brains of *Sidt2*^−/−^ mice, the majority of which developed seizures with age. Other mouse studies involving loss of SIDT2 indicate that the protein is also important for lysosomal function in other organs. For example, lysosomes perform a key degradative role for all types of autophagy, and various studies have now observed defective autophagy across a range of tissues in *Sidt2*^−/−^ mice, including liver [7], kidney [8] and skeletal muscle [9].

LSDs most commonly arise due to defects in genes encoding lysosomal enzymes, but mutations in lysosomal transporters and membrane proteins have also been implicated. As noted earlier, SIDT2 is a lysosomal membrane protein, but it has also been reported to have enzymatic and transporter functions. For instance, recent studies indicate that SIDT2 forms a tightly packed dimer with a Zn^2+^-dependent catalytic core and has ceramidase activity, consistent with previous observations that loss of SIDT2 leads to triglyceride and lipid droplet accumulation in the liver [7]. Previously, another group reported that SIDT2 conducts sodium ions and might play a role in the H^+^-ATPase counter ion flux in lysosomes [10]. Meanwhile, we have demonstrated that SIDT2 in mice transports double-stranded RNA (dsRNA) into the cytoplasm [11], a role consistent with that of its C. elegans ortholog, SID-1, and recent reports in humans [12]. Others have described a more widespread role in nucleic acid transport, involving the passage of both DNA and RNA into lysosomes [13,14]. Consistent with an RNA transporter function, SIDT2 has previously been reported to play an important role in RNautophagy [13,15] a process believed important for lysosomal destruction of RNA. The only other protein thus far implicated in RNautophagy is the LAMP2 isoform, LAMP2C. Mutations in LAMP2 cause Danon disease [16,17], a rare LSD that affects multiple organ systems, including the brain, heart and skeletal muscle.

Taken together, we therefore hypothesized that loss of SIDT2 in mice might result in changes similar to those seen in humans with Danon disease, and focused our attention here on skeletal muscle. After all, muscle-specific deletion of *Sidt2* in mice has been previously observed to cause reduced muscle mass and strength, in association with evidence of muscle damage (e.g., increased serum creatine kinase and central nucleation of muscle fibers) and autophagic dysfunction [9,18] (e.g., accumulation of p62 and LC3-II, reduced clearance of damaged mitochondria). However, explicit links between loss of SIDT2 and Danon disease have not previously been investigated. Using a global *Sidt2* knockout mouse model, we therefore investigated these links, while at the same time exploring the underlying physiological and cellular basis via which SIDT2 impacts skeletal muscle function.

## 2. Results

### 2.1. Loss of SIDT2 Leads to Reduced Muscle Mass and Strength in Association with Fiber Vacuolation and Impaired Autophagy

**Figure 1:**
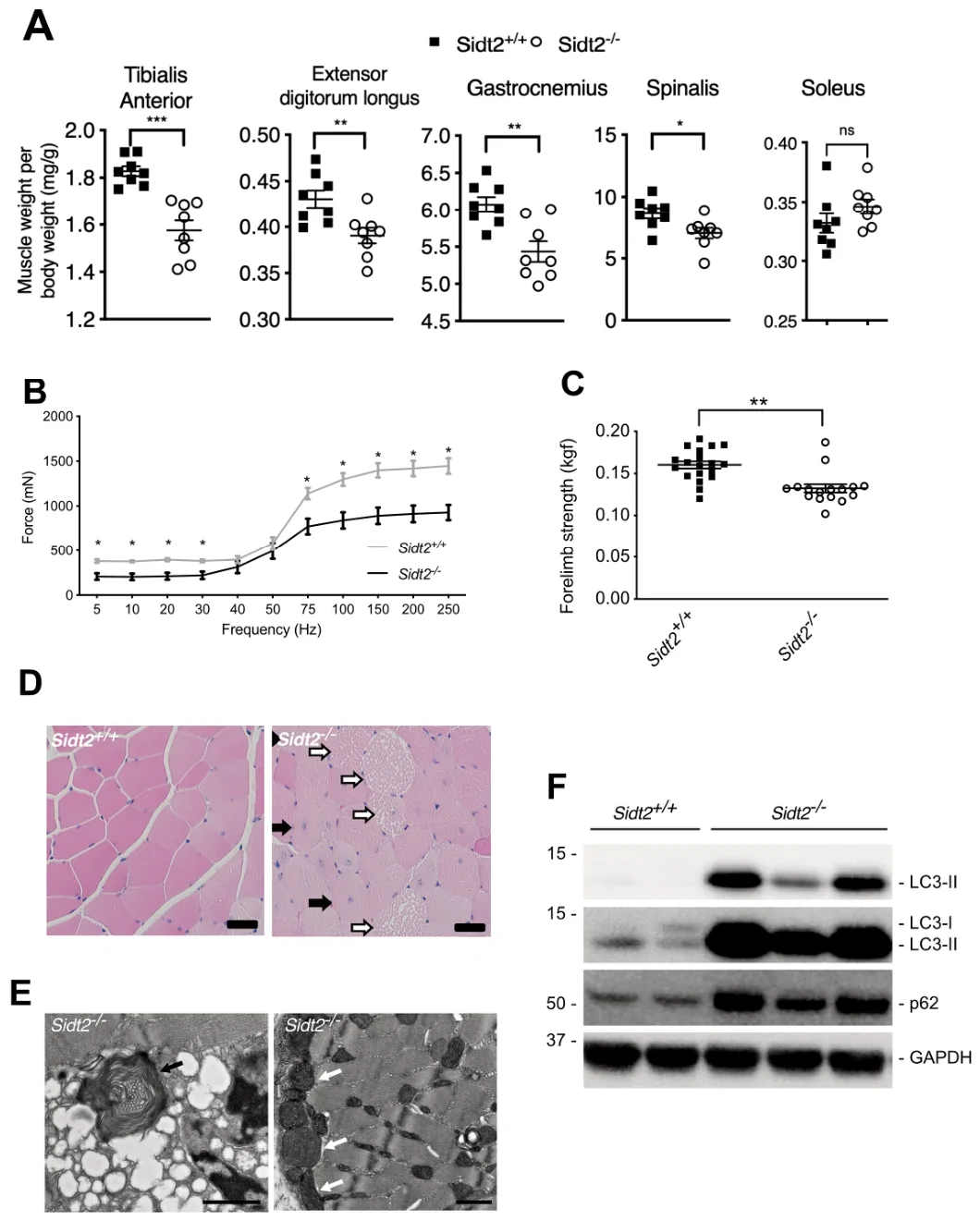
Loss of SIDT2 leads to reduced muscle mass and strength in association with fiber vacuolation and impaired autophagy. (**A**) Indicated muscles from 8-week-old Sidt2^+/+^ and Sidt2^−/−^ mice (*n* = 8) were isolated, weighed and plotted relative to total body weight of each animal. Each symbol represents an individual mouse, and mean ± SEM are shown. Data were compared using a Student’s *t*-test (* *p* < 0.05, ** *p* < 0.01, *** *p* < 0.0001, ns, not significant). (**B**) Comparison of absolute force from tibialis anterior muscle fibers from 12-month-old Sidt2^+/+^ and Sidt2^−/−^ mice. Error bars = SE. (**C**) Grip strength of 8–10-months-old male mice. Each symbol represents an individual mouse, and mean ± SEM are shown. (**D**) H&E-stained sections of formaldehyde fixed, paraffin-embedded tibialis anterior from Sidt2^+/+^ and Sidt2^−/−^ mice. Myofibers from the latter display vacuolation (white arrows) and central nuclei (black arrows), a hallmark of regenerating myofibers. Scale bar: 25 μm. (**E**) Electron microscopy of Sidt2^−/−^ quadriceps muscle shows numerous vacuoles, occasional multi-membranous “myelin figures” (black arrows) and abnormal mitochondria (white arrows). Scale bar: 1 μm. (**F**) Western blotting for LC3 and p62 in quadriceps from Sidt2^+/+^ and Sidt2^−/−^ mice shows an increase in both these proteins. GAPDH is provided as a loading control.

To begin, we found evidence of significantly reduced muscle mass in Sidt2^−/−^ animals from as young as 8 weeks of age across all tested muscle groups (tibialis anterior, extensor digitorum longus, gastrocnemius, spinalis) with the exception of soleus (Figure 1A). Next, we performed electrophysiology experiments on tibialis anterior muscle in anesthetized mice, and observed that Sidt2^−/−^ mice generated significantly less absolute force compared to wild-type animals (Figure 1B). We then compared the grip strength of Sidt2^+/+^ and Sidt2^−/−^ mice as a measure of the maximal force that can be generated in the forelimbs of the animals (Figure 1C), and observed that grip strength of Sidt2^−/−^ mice was significantly weaker than wild-type mice.

To understand these muscle changes in Sidt2^−/−^ mice at the cellular level, we stained the myofibers with H&E and observed that SIDT2^−/−^ myofibers had central nuclei and abnormal vacuolation (Figure 1D). Similarly, electron microscopy showed multiple atypical features, including abnormal vacuoles and enlarged mitochondria (Figure 1E). Finally, we also observed an accumulation of LC3-II and p62 protein in skeletal muscle from Sidt2^−/−^ mice (Figure 1F).

Taken together, these results confirmed previous reports of the changes that arise due to deletion of Sidt2 in mouse skeletal muscle [9,18].

### 2.2. Skeletal Muscle from Sidt2^−/−^ Mice Resembles Changes Seen in Danon Disease

**Figure 2:**
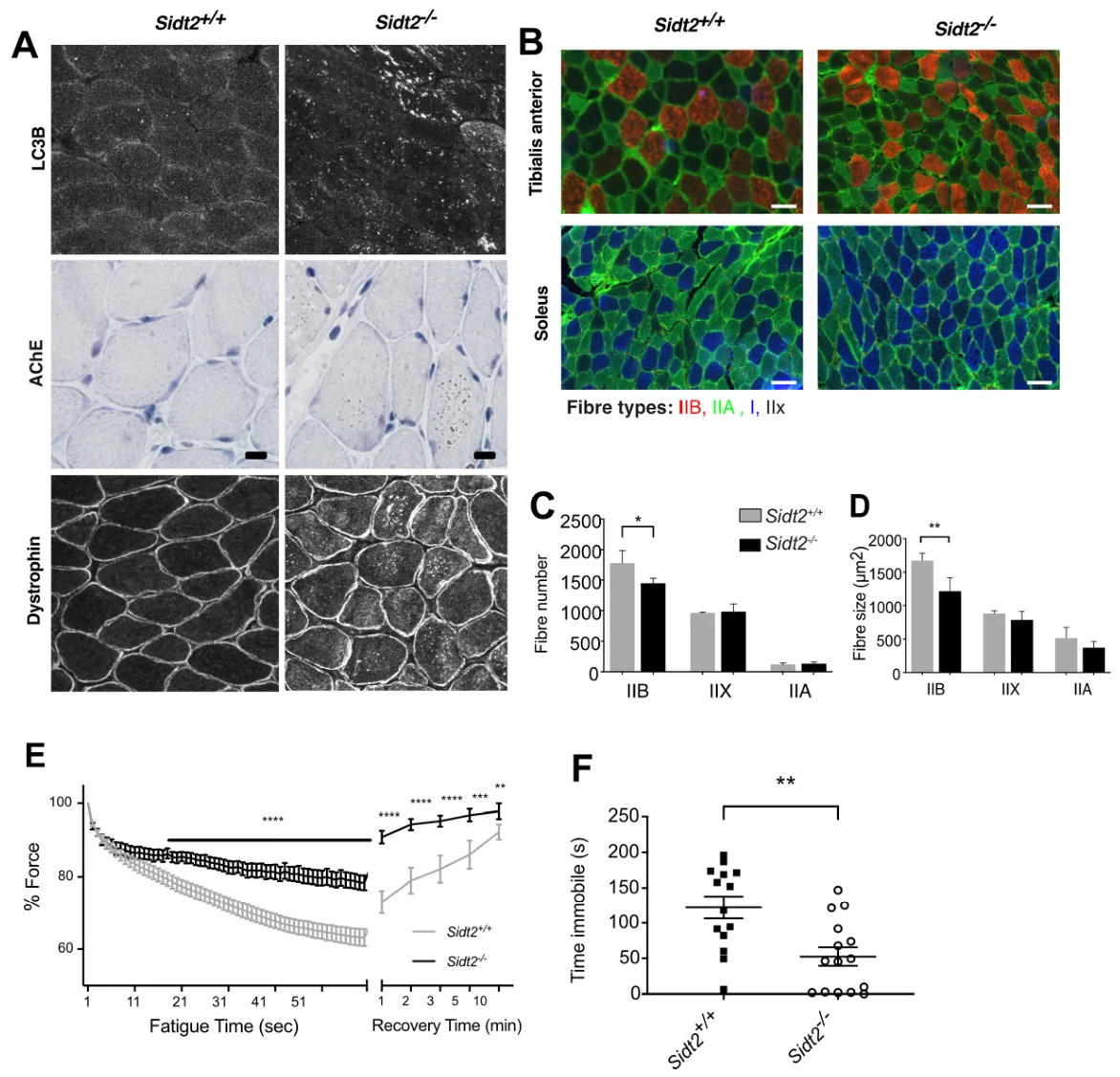
Skeletal muscle from *Sidt2*^−/−^ mice resembles that of humans with Danon disease. (**A**) Transverse sections of soleus muscle from 8-week old Sidt2^+/+^ and Sidt2^−/−^ mice were assessed for LC3 by immunostaining (top panel), acetylcholinesterase activity by histochemistry (middle panel), and dystrophin by immunostaining (bottom panel). In the absence of SIDT2, each of these features is observed within vacuolar structures. Scale bar: 10 μm. (**B**) Representative images from 8-to-10-week-old SIDT2^+/+^ and Sidt2^−/−^ mice of tibialis anterior (top panels) and soleus (bottom panels) muscle sections immunostained for different fiber types: red (IIB), green (IIA), blue (I), black (IIx). Scale bar: 50 μm. (**C**,**D**) Individual fiber size and count of different muscle fiber types in Sidt2^+/+^ and Sidt2^−/−^ mice. Data were compared using a Student’s *t*-test (* *p* < 0.05, ** *p* < 0.01). To account for multiple testing, Holm–Šídák correction was applied with a family-wise significance level (α) of 0.05. (**E**) Fatigue recovery of tibialis anterior fibers from 12-month-old SIDT2^+/+^ and Sidt2^−/−^ mice. Error bars = SE; n = 7 SIDT2^+/+^, n = 9 Sidt2^−/−^. Data were compared using multiple *t*-test and 2-way ANOVA where relevant (** *p* < 0.01, *** *p* < 0.0001, **** *p* < 0.00001). (**F**) Forced swim test performance of 2–3 month old Sidt2^+/+^ and Sidt2^−/−^ mice, showing the time spent immobile during the 6 min test. Each symbol represents an individual mouse. Mean ± SEM are shown. Error bars = SE; n = 14 SIDT2^+/+^, n = 15 Sidt2^−/−^. Data were compared using ordinary ANOVA (** *p* < 0.01).

To investigate whether loss of SIDT2 in mice might result in changes similar to those seen in humans with Danon disease, we first assessed Sidt2^−/−^ mice for one of the key pathognomonic features of Danon disease, namely the presence of autophagic vacuoles with sarcolemmal features (AVSFs) in skeletal muscle [16]. Consistent with the presence of AVSFs, we noted the accumulation of LC3, acetylcholinesterase activity and dystrophin in a vacuolar distribution within the skeletal muscle of Sidt2^−/−^ animals (Figure 2A).

Although Danon disease is known to affect both type I (slow-twitch, oxidative, fatigue-resistant) and type II (fast twitch, glycolytic) skeletal muscle fibers, selective atrophy of type II fibers was recently reported [19]. To investigate whether this might also be true in Sidt2^−/−^ mice, we immuno-stained sections of tibialis anterior muscle to identify the different fiber types present in mouse skeletal muscle, namely type I (slow-twitch, oxidative), IIa (fast twitch, oxidative), IIx (intermediate), and IIb (fast twitch, glycolytic) fibers. Using a previously published automated computational analysis method [18], thousands of individual fibers were then assessed in terms of both their size and fiber type. By doing so, we observed a significant decrease in both the size and number of type IIb fibers in Sidt2^−/−^ mice (Figure 2B–D). In contrast, type IIa and IIx fibers showed no difference, while type I fibers were not present in sufficient number to be analyzed, in keeping with previous analyses of tibialis anterior muscle. Taken together, these data suggested that loss of SIDT2 specifically affects fast twitch, glycolytic fibers, and were consistent with our inability to observe any weight difference in soleus (Figure 1A), the prototypic slow twitch muscle in mice [20]. To test the functional impact of these changes, we next compared fatigue and recovery of tibialis anterior fibers via electrophysiology (Figure 2E) and observed reduced fatigue and enhanced recovery in Sidt2^−/−^ mice. We then compared Sidt2^+/+^ and Sidt2^−/−^ mice in a forced swim test as a measure of endurance and observed that Sidt2^−/−^ mice continued swimming for longer than wild-type mice (Figure 2F).

**Figure A1:**
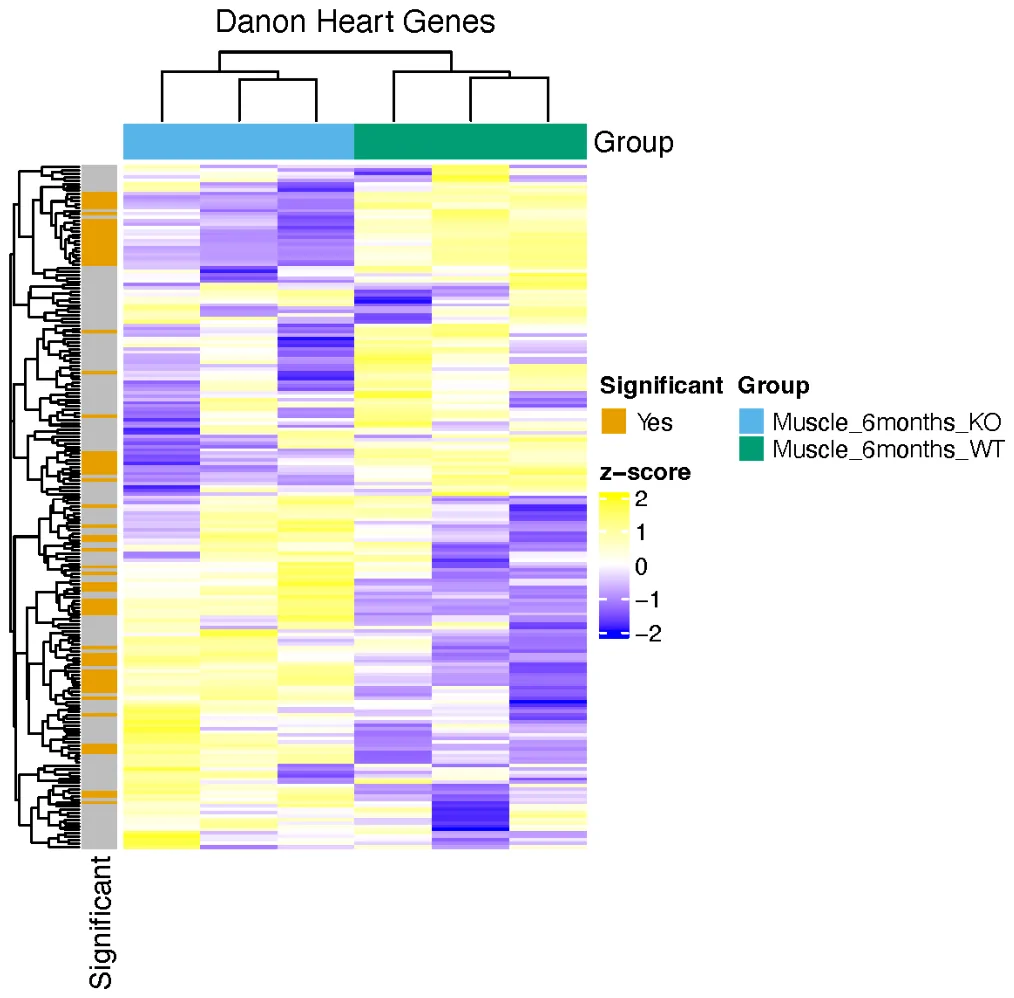
A heatmap showing expression of the differentially expressed gene set from Danon disease heart samples in the Sidt2 KO vs. WT mouse RNA-seq data set. Values are log2-reads per kilobase per million mapped reads (RPKM), scaled and centered by gene (row). Genes highlighted in orange are those that are significant when comparing Sidt2 KO to WT samples at an FDR of 0.05.

Finally, to further explore possible commonalities between the loss of SIDT2 and Danon disease, we undertook an RNAseq analysis of Sidt2^+/+^ and Sidt2^−/−^ skeletal muscle. We then compared the approximately 1000 genes that were differentially expressed in the absence of Sidt2 with genes dysregulated in Danon disease. For the latter, we performed RNAseq on bio-banked heart tissue from Danon disease patients and healthy individuals (n = 3/group). By mapping the 57 differentially expressed genes from Danon disease patients onto corresponding mouse genes and then performing ROAST gene set analysis [21] with genes differentially expressed in the absence of Sidt2, we observed that the expression signature of Danon disease in the human patients was significantly correlated with the expression signature of the Sidt2^−/−^ mouse samples (*p*-value = 0.0001) (Appendix A Table A1 and Figure A1). Consistent with this, many significantly enriched gene ontology terms (GO terms) were common across the two experiments, including not only expected biological processes such as muscle cell development and autophagy, but also cell death and apoptosis.

Taken together, these data indicate that loss of SIDT2 in mouse skeletal muscle closely resembles LAMP2 deficiency in Danon disease patients.

### 2.3. Loss of Sidt2 Leads to Increased Expression of linc-MD1, Induction of a Type I Interferon Response, and Upregulation of Cell Death

**Figure 3:**
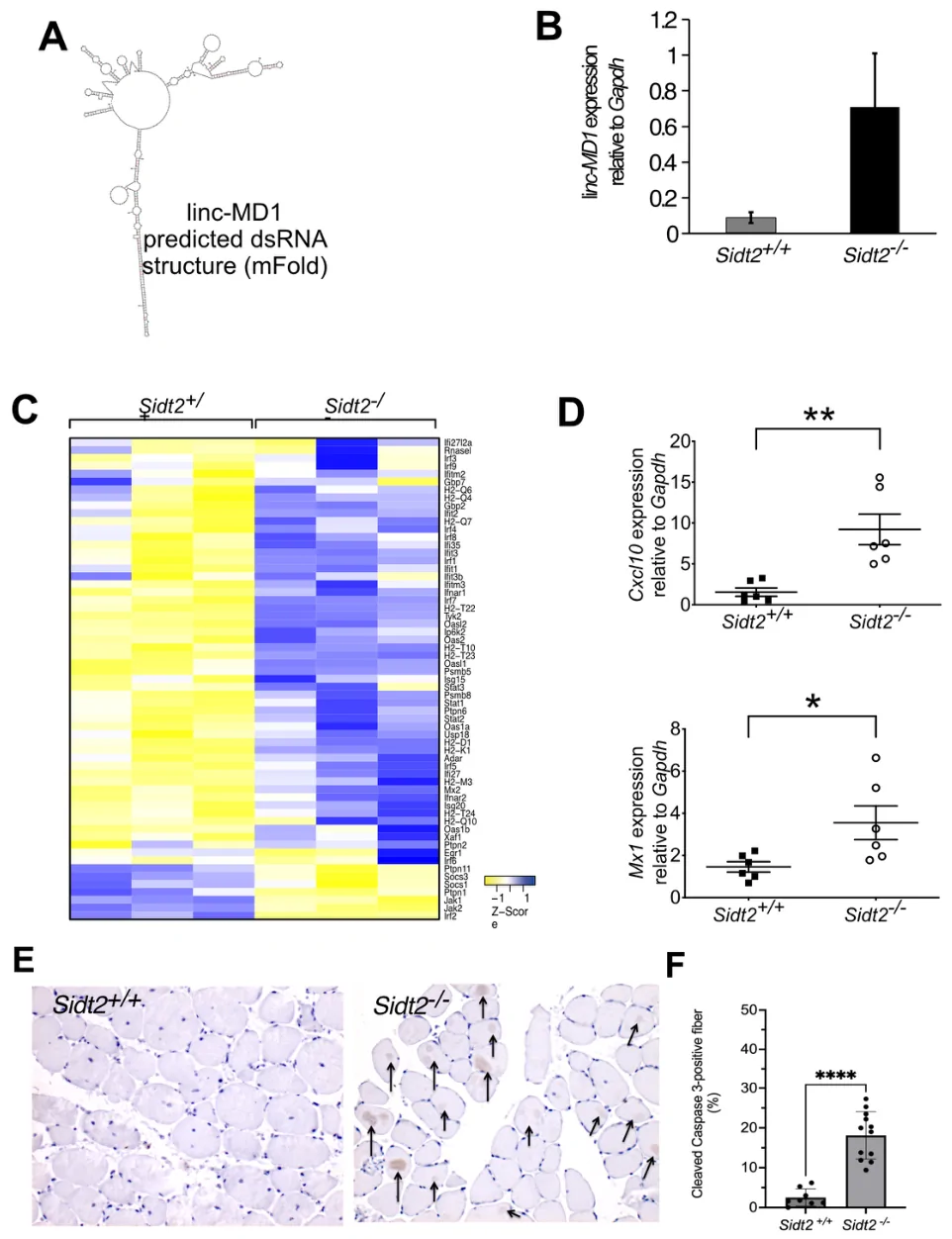
Loss of Sidt2 leads to induction of type I interferon response, high expression of dsRNA in muscles and cell death. (**A**) Predicted dsRNA structure of lincMD-1. (**B**) Linc-MD-1 expression in quadriceps muscles of Sidt2^+/+^ and Sidt2^−/−^ mice as analyzed by qRT-PCR; error bars = SE. (**C**) Heatmap showing significant enrichment of genes involved in interferon alpha beta signaling in Sidt2^−/−^ mice. (**D**) qRT-PCR analysis showing significant increase in relative expression of CXCL-10 and MX1 of Sidt2^−/−^ muscles; n = 6, error bars = SEM, * *p* < 0.05, ** *p* < 0.01, Student’s *t*-test. (**E**) Quadricep muscle sections of 6-month-old Sidt2^+/+^ and Sidt2^−/−^ mice were stained with cleaved Caspase 3 antibody; black arrows indicate cleaved caspase 3-positive fibers. (**F**) Percentage of cleaved-caspase 3-positive fibers; error bars = SEM. **** *p* < 0.0001, Student’s *t*-test.

To better understand the cellular changes that occur following loss of SIDT2, we further examined our RNAseq data from Sidt2^+/+^ and Sidt2^−/−^ skeletal muscle for clues. Within the top 20 most upregulated genes in Sidt2^−/−^ skeletal muscle (Table A2) was a long noncoding RNA, linc-MD1, which was previously implicated as being important in muscle cell development [22]. Given the reported roles for SIDT2 in dsRNA transport and RNA autophagy [13,15], we were interested to note that this long noncoding RNA is predicted to fold into a long dsRNA based upon secondary structure analysis (Figure 3A), and we subsequently confirmed via qRT-PCR that linc-MD1 is strongly upregulated (>30-fold) in Sidt2^−/−^ muscle (Figure 3B).

We then performed gene set enrichment analysis (GSEA) on the RNASeq data to determine the molecular pathways affected by loss of SIDT2. Consistent with the increased levels of linc-MD1 RNA and the role of dsRNA in activating the innate immune response, we found that the type I interferon signaling pathway showed significant differences between Sidt2^+/+^ and Sidt2^−/−^ skeletal muscle (*p* = 2.75 × 10^−8^) (Figure 3C). These results were later validated via qRT-PCR for canonical interferon-stimulated genes (Figure 3D).

A shared consequence of both increased type I IFN signaling and impaired autophagy is cell death via apoptosis. Based on the evidence of increased type I IFN signaling and impaired autophagy in Sidt2^−/−^ muscle, we hypothesized that there might be elevated rates of apoptosis in these tissues. To assess this possibility, muscle sections from Sidt2^+/+^ and Sidt2^−/−^ animals were immune-stained for cleaved caspase 3, which is a key effector protein involved in mediating apoptosis. Sidt2^−/−^ mice displayed a significantly increased proportion of muscle fibers positive for cleaved caspase 3 compared to controls (Figure 3E,F) indicating that loss of SIDT2 is indeed associated with increased apoptosis in skeletal muscle.

## 3. Discussion

Although many different lysosomal membrane proteins have been identified, SIDT2 and LAMP2 are the only ones to have been implicated in RNAutophagy. Here, we report that loss of SIDT2 in mice results in various changes in skeletal muscle that also arise due to LAMP2 dysfunction in Danon disease. Taken together with our recent observation that loss of SIDT2 activity in both humans and mice also results in neurological dysfunction—which is also a core feature of Danon disease—it thus appears that SIDT2 and LAMP2 share various common aspects in their biology. Indeed, individuals with Danon disease and/or animals lacking LAMP2 have been observed to have liver, kidney and pancreatic dysfunction [23,24], all of which have also been identified in Sidt2^−/−^ animals.

**Figure A2:**
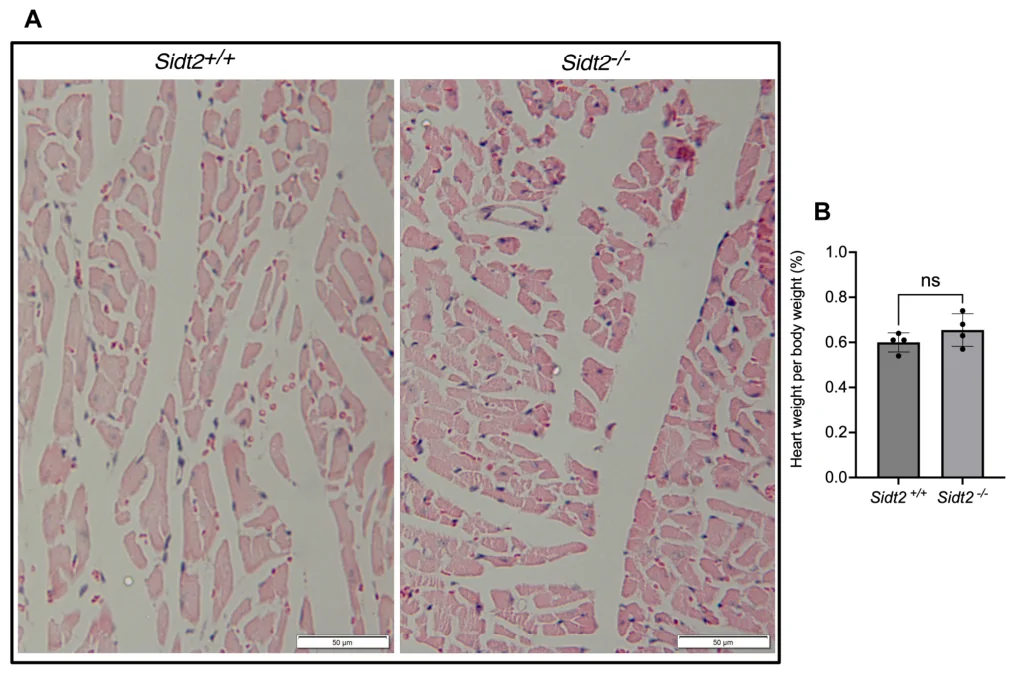
(**A**) H&E-stained sections of formaldehyde fixed, paraffin-embedded cardiac muscle from Sidt2^+/+^ and Sidt2^−/−^ mice. Scale bar: 50 μm. (**B**) Comparison of heart weights between 10-week-old Sidt2 wild-type and Sidt2 knockout mice. Data are presented for n = 4 mice per group. Statistical comparisons were performed using an unpaired two-tailed Student’s *t*-test, and *p* was > 0.05, ns, not significant.

One apparent divergence, however, is that both patients with Danon disease as well as Lamp2^−/−^ mice typically develop cardiomyopathy, features of which have not been observed in our Sidt2^−/−^ mice, whose heart size and histology was similar to those of wild-type animals (Appendix A, Figure A2) nor reported by others who have characterized mice with a global knockout of Sidt2 [25,26]. One possible explanation for this is that SIDT2 appears to be poorly expressed in the heart [4,25]. Another possibility is that mutations in LAMP2 may involve loss of not only LAMP2C function—which is the specific isoform that has been implicated in RNAutophagy—but also the function of the other two LAMP2 isoforms, LAMP2A and LAMP2B, and these other isoforms might be more important in maintaining normal cardiac function.

The mechanism by which SIDT2 leads to lysosomal dysfunction is still unclear but various possibilities exist. One relatively simple explanation is that SIDT2—as a large lysosomal transmembrane protein—may play an important structural role during autophagy. For example, lysosomal transmembrane proteins have been proposed to regulate autophagy by controlling lysosomal motility and fusion with autophagosomes [27]. Another possibility is that SIDT2—consistent with a recent proposal that it may function as a cation-conducting channel [10,28] and in keeping with an earlier study which reported that loss of SIDT2 hampers calcium efflux [29]—may help to maintain lysosomal acidification (which is crucial to the function of its many degradative enzymes) by promoting a cation counter-flux to balance the import of protons into the lysosomal lumen [30]. Third, as mentioned earlier, SIDT2 has recently been reported to have ceramidase activity, and disruptions in the function of lysosomal enzymes is a well-known cause of lysosomal storage disorders. Finally, a novel possibility and one consistent with SIDT2’s known role in RNA (and potentially DNA) transport is that the import of negatively charged nucleic acids by SIDT2 into the lysosome (as part of RNautophagy, for example) provides an anion influx that supports lysosomal acidification and function. Related to this, despite multiple reports supporting a role for SIDT2 in nucleic acid transport, the identity of any endogenous nucleic acid substrates remains unclear. Our RNA-Seq results provided one candidate—linc-MD1—although it would seem unlikely if this were the only RNA substrate for SIDT2. Recently, Guo and colleagues reported that autophagy helps to maintain genome stability by degrading retrotransposon RNA from both long (LINE-1) and short interspersed (Alu) elements [31]. It would therefore be interesting in the future to test whether SIDT2 might be involved in the transport of these RNAs (especially Alu elements which form dsRNAs via their inverted repeats). To this end, unbiased identification of the RNA species with which SIDT2 interacts (e.g., via RNA immunoprecipitation and sequencing) would be highly informative.

Mechanistically, another question that deserves consideration is how loss of SIDT2 leads to the muscle changes observed by us and others. Potentially both impaired autophagy and induction of type I IFN signaling could independently drive the apoptosis observed in Sidt2^−/−^ muscles. Certainly, muscle disease is a feature of type I interferonopathies—as exemplified in human diseases such as Aicardi–Goutières syndrome—and while this typically includes muscle atrophy similar to that seen in our Sidt2^−/−^ mice, muscle histology in such cases characteristically includes inflammatory infiltrates [32] which were absent in our mice. In contrast, mice in which core components of the autophagy pathway have been conditionally deleted in skeletal muscle more closely resemble Sidt2^−/−^ mice. Specifically, muscle-specific deletion of the critical autophagy gene, Atg7, leads to not only reduced myofiber size, muscle atrophy and reduced contractile force, but also formation of multilamellar vacuolar structures [33] and accumulation of LC3 and p62 as seen in our mice. Similarly, in the context of human disease, accumulation of autophagic vacuoles is believed to be the primary driver of myofiber breakdown and muscle weakness [34]. Autophagic dysfunction [35] is also known to preferentially impact fast twitch, glycolytic fibers, which were significantly decreased in both size and number in our Sidt2^−/−^ mice and were presumably the source of the increased apoptosis we observed (although this would need to formally tested as a future direction). Of course, it is also possible that neither impaired autophagy nor induction of type I IFN signaling are the primary pathogenic drivers in Sidt2^−/−^ mice. For example, Gao and colleagues previously reported that loss of SIDT2 leads to ER stress [36] within the liver, and ER stress can induce both apoptosis [37] and autophagy [38]. However, given that impaired autophagy can induce ER stress by increasing the unfolded protein response, it is also possible that the ER stress observed in this previous study was secondary to a primary defect in autophagy.

It is also important to acknowledge a number of important limitations in our study. First, although the concurrent upregulation of linc_MD1, type I interferon signaling and apoptosis is intriguing, the present data do not establish a causal relationship between these observations. Further studies will thus be required to determine whether linc-MD1 contributes to the activation of interferon signaling and apoptosis in Sidt2^−/−^ skeletal muscle (e.g., via experiments that explicitly assess for dsRNA accumulation and employ loss- or gain-of-function approaches). Second, although the expression signature of Danon disease cardiac muscle was significantly correlated with the expression signature of Sidt2^−/−^ mouse skeletal muscle, we did not detect evidence of a type I interferon pathway activation in the Danon disease samples. Whether this was due to intrinsic differences in skeletal and cardiac muscle or to the limited power of our pathway analyses in the Danon samples (for which there were only 57 differentially expressed genes) is unclear. Future well-powered studies looking for type I interferon activation in both cardiac and skeletal muscle from Danon disease patients and Lamp2^−/−^ mice would thus be valuable. If patients with Danon disease were found to have increased type I interferon signaling, this would open up the possibility of using existing strategies to block or dampen type I IFN signaling (e.g., Anifrolumab, Dazukibart, JAK inhibitors) as a therapeutic modality for Danon disease. Third, we acknowledge that our assessment of apoptosis in Sidt2^−/−^ muscle was limited to cleaved caspase 3 immunostaining. This was despite our attempts to supplement these data with cleaved caspase 3 Western blots and TUNEL staining, which proved technically challenging.

Finally, looking ahead, there are other avenues for further study apart from those already mentioned above. For example, generation of Sidt2^−/−^ Lamp2^−/−^ mice and phenotypic comparison to single knockout animals could provide insights into epistasis and functional redundancy. In addition, retinopathy and intellectual disability are common features of Danon disease and assessing Sitd2^−/−^ mice for their retinal structure and function as well as learning and memory might be informative.

## 4. Materials and Methods

### 4.1. Mice

Mice were bred and maintained in the animal facilities at the Walter and Eliza Hall Institute of Medical Research (WEHI) and at the Department of Molecular and Cellular Biology, Harvard University, according to national and institutional guidelines for animal care. Mice were housed in individual ventilated cages at 19–24 °C and maintained on a 14 h light to 10 h dark cycle with continuous access to Barastoc Custom Mixed Ration (irradiated) and acidified and filtered water. All experimental procedures were approved by the relevant animal ethics committees at the WEHI and Harvard University. Targeted Sidt2 alleles were generated as part of a mouse knockout library for transmembrane proteins as described previously [38] and mice were obtained from the Knock-Out Mouse Project (KOMP) Repository (www.komp.org). These mice were originally on a 129 background but were subsequently backcrossed at least 6 times onto a C57/Bl6 background.

### 4.2. Muscle Harvest, Histology and Staining

Mice were euthanized by cervical dislocation immediately prior to tissue collection. Quadriceps, tibialis anterior, spinalis, extensor digitorum longus and gastrocnemius muscles were removed, weighed where indicated, and either snap frozen in liquid nitrogen for protein or RNA analysis or frozen in OCT medium following cooling in isopentane for histological analysis.

For H&E staining, relevant skeletal muscle samples were suspended in 4% paraformaldehyde prior to sectioning and staining. For immunostaining analysis, five micron transverse sections were obtained from midsection of tibialis anterior or quadriceps muscles and then stained as described previously [39]. To identify different fiber types, fiber borders were stained with wheat germ agglutinin and tissues were stained with myhc antibodies against type 1 myosin heavy chain (Chemicon), type IIB (DSHB, University of Iowa), and IIA (DSHB, University of Iowa), and dystrophin (dys6-10; L. Kunkel, Boston Children’s Hospital, Harvard Medical School, Boston, MA, USA). Images were captured using an Olympus BX50 microscope (Hachioji City, Tokyo, Japan) and analysed using ProgRes software (SciTech Pty Ltd, Preston, Victoria, Australia). Each type of fibers per muscle section was counted and quantified using Meta-Morph Software v7.8 (Molecular Devices, Sunnyvale, California). Fiber diameters were measured using ImagePro 2.0 software (Media Cybernetics, Silver Spring, MD, USA). Fiber type proportions were determined as the percentage of positive fibers of the total fibers in a given field.

For detection of cleaved caspase 3, paraffin-embedded sections of 4% paraformaldehyde-fixed tissues were cut onto silane-coated slides. Cleaved caspase 3 immunohistochemistry was performed on a Dako Omnis automated staining platform (Glostrup, Denmark). Following deparaffinization and high-pH antigen retrieval (EnVision FLEX TRS, Glostrup, Denmark, 40 min), sections were incubated with anti-cleaved caspase 3 (Asp175; Cell Signaling Technology, Danvers, MA, USA, #9661; 1:300). Images were captured using an Olympus IX70 inverted Fluorescent Microscope (Olympus, Tokyo, Japan).

### 4.3. Transmission Electron Microscopy (TEM)

TEM analysis of skeletal muscle samples was performed by fixing muscle samples in 2.5% glutaraldehyde in 0.1 M PBS, pH 7.4, overnight at 4 °C. Washing was done with PBS and then tissues were suspended in 1% OsO4 for 2 h at room temperature. Finally, tissues were dehydrated in acetone, embedded in Epon 812, sectioned, and imaged on a JEOL 1400 electron microscope (Akishima, Tokyo, Japan) with Gatan Orius CCD cameras.

### 4.4. Grip Strength Measurement

Forearm grip strength of the mice was measured by lifting the mice by the tail and then allowing them to grasp the bar of a grip strength meter (Columbus Instruments, Columbus, OH, USA) as described previously [40]. The mouse was then pulled backwards away from the bar while keeping its body in a straight horizontal line. Value of peak force from the meter display was recorded and the trial was repeated ten times. All mice were tested by an operator blind to the genotype.

### 4.5. Absolute Force and Fatigue Measurement

Following animal anesthetization with isoflurane, the tibialis anterior (TA) muscle was assessed in situ using the Arora Scientific 1300A (Aurora Scientific Inc., Aurora, ON, Canada) whole mouse test system and 701C stimulator as previously described [41]. Specifically, the distal tendon of the TA was isolated and suture silk was used to secure the tendon to the force transducer (Model 809B in situ test apparatus), while the foot and knee were fastened in place. The TA muscle was adjusted to an optimum length to produce maximum twitch force (Pt), and the fiber length was calculated using a correction value of 0.6. The TA was then stimulated every 2 min at increasing frequencies (5–250 Hz) to generate force–frequency curves. Both optimum length and TA mass were recorded and used to determine the maximum twitch force for each muscle. After a 10 min rest, muscles were subjected to a fatigue protocol, which involved repeated 1 s tetani at 100 Hz every 2 s for a period of 30 s. To track recovery, the force produced by short (250 ms) 100 Hz tetani was recorded at 30 s, 1, 2, 3, 5 and 10 min post fatigue. The force produced during each of the 100 Hz tetani and subsequent recovery was recorded and expressed relative to the pre-fatigue force.

### 4.6. Forced Swim Test

Mice were transported to a separate room for testing. A 1 L beaker was filled with 500 mL water at 25 °C, and mice were deposited into the beakers, where they were free to float, swim, or paw at the sides of the beaker. Each mouse was tested for 6 min, where the first 2 min were designated as pre-test and the last 4 min as the test. Each test was recorded using a video camera. The time that the mouse kept paddling in the water and the time it was immobile were recorded. After the test, the mouse was placed in a drying cage containing paper towel, with a heat lamp over it and a heat pad underneath. Fresh tap water was used for each mouse.

### 4.7. Western Blot

Frozen quadriceps muscles were lysed in a buffer containing 20 mM Tris (pH 7.5), 135 mM NaCl, 1.5 mM MgCl2, 1 mM EDTA, 10% glycerol, 1% Triton X-100 supplemented with protease and phosphatase inhibitor (Sigma, MO, USA) and homogenized usingTissueLyser II (Quiagen, Hilden, Germany). Homogenates were then spun down at 13,000× *g* at 4 °C for 15 min. Total protein was quantified using Pierce BCA Protein Assay kit (Thermofisher Scientific, Rockford, IL, USA). Measurements of 40–50 micrograms of protein per lane were loaded onto 4–12% NuPAGE precast gels (Invitrogen, Carlsbad, CA, USA), separated by SDS-PAGE, and transferred to polyvinylidene fluoride membranes (Millipore, County Cork, Ireland) at 100 voltz for an hour. Membranes were then blocked with 5% commercial BSA in 1× TBS, 0.1% Tween 20 for 30 min and then probed with the following antibodies overnight at 1:1000 dilution: LC3B (D11, Cell Signaling Technologies), p62 (#5114, Cell Signaling Technologies) and GAPDH (#MAB374, Merck, Danvers, MA, USA) Blots were then washed three times for 10 min, probed with anti-rabbit secondary-HRP antibody for an hour, washed as before then treated with Luminata Forte Western HRP substrate (Millipore), and visualized using the ChemiDoc MP system (Bio-Rad, Hercules, CA, USA).

### 4.8. RNA Isolation

Total RNA was extracted from 6-month muscle tissues using Isolate II RNA Mini Kit (Bioline, London, UK) as per the manufacturers’ instructions. RNA yield was quantified using NanoDrop (NanoDrop Technologies, LLC, Wilmington, DE, USA) and RNA quality was assessed using either the TapeStation System 2200 or the Bioanalyser (Agilent Technologies, Santa Clara, CA, USA). To remove contaminating DNA, 1 μg RNA was treated with 1 μL (2U) of DNAse I (Ambion, Austin, TX, USA) in a 20-μL reaction incubated at 37 °C for 30 min and 1 μL DNAse inactivation reagent added.

### 4.9. RNA-Seq

A measurement of 500 ng of DNAse-treated RNA (isolated using Tri-Reagent) was used as a starting material for RNA-Seq. RNA sequencing libraries were prepared using Illumina TruSeq (San Diego, CA, USA) stranded total RNA library prep kit and sequenced on an Illumina NextSeq 500, generating 80bp paired-end reads. All samples were aligned with the mm10 build of the mouse genome using the Rsubread aligner (Melbourne, Australia) [42]. The number of reads overlapping Entrez genes was summarized using featureCounts and NCBI RefSeq annotation. Differential expression analyses were then performed using edgeR and limma [43,44].

All genes which did not achieve a CPM greater than 1 in at least 3 samples were filtered from the data as they were considered to be unexpressed. Additionally, genes with no current symbol and sex-linked genes (Xist and Y chromosome) were also removed. Compositional differences between samples were normalized using the TMM method. All counts were then transformed to log2CPM with associated precision weights using voom [45]. Differential expression between the sample groups was assessed using linear models and robust empirical Bayes moderated t-statistics [46]. The *p*-values were adjusted to control the FDR below 5% using the Benjamini and Hochberg method.

### 4.10. qRT-PCR

One microgram of DNAse-treated RNAs was reverse transcribed using Superscript II reverse transcriptase (Invitrogen, Carlsbad, CA, USA) and oligo(dT)15 primers (Promega, Fitchburg, WI, USA) according to the manufacturers’ recommendations. Real-time PCR was done with 2x AccuPower Greenstar qPCR Mastermix (Bioneer Pacific, Daejeon, South Korea) and run on a LightCycler 480 (Roche, Rotkreuz, Switzerland). GAPDH was chosen as the housekeeping gene because its expression was found to be consistent between Sidt2^+/+^ and Sidt2^−/−^ muscles by Western blot while B-actin levels were variable. The following primer sequences were designed and used in the study, which were synthesized by Bioneer Pacific: Cxcl-10 (Fwd: GCCGTCATTTTCTGCCTCA; Rev: CGTCCTTGCGAGAGGGATC); GAPDH (Fwd: CAACTTTGTCAAGCTCATTTCCTG; Rev: CCTCTCTTGCTCAGTGTCCTT); linc-MD1 (Fwd: GCAAGAAAACCACAGAGGAGG; Rev: GTGAAGTCCTTGGAGTTTGAG) and Mx-1 (Fwd: GATCCGACTTCACTTCCAGATGG; Rev: CATCTCAGTGGTAGTCAACCC).

### 4.11. Danon Disease Studies

Left ventricular tissue samples from patients with end-stage heart failure due to Danon disease were collected at the time of cardiac transplant, while control samples were obtained from donor hearts that were procured for transplantation but then unused for non-cardiac reasons. All tissue samples were flash frozen immediately in liquid nitrogen in the operating room (without the use of any cardioplegia solutions) and transported to a −80 °C freezer where they were stored until use.

### 4.12. Statistics

Unless otherwise indicated, statistical analyses were performed using GraphPad Prism version 10.0 (GraphPad Software, San Diego, CA, USA). Differences between two groups were assessed using unpaired, two-tailed Student’s *t*-tests. Data are presented as mean ± SEM unless otherwise specified, and *p* < 0.05 was considered statistically significant.

To account for multiple comparisons in fiber-type analyses, the Holm–Šídák correction was applied using a family-wise significance level (α) of 0.05. Fatigue recovery data were analyzed using multiple *t*-tests with correction for multiple testing and two-way ANOVA where appropriate. Forced swim test data were analyzed using ordinary one-way ANOVA. Details of the statistical analyses performed for each experiment are provided in the relevant figure legends.

## 5. Conclusions

Our findings identify SIDT2 as a critical regulator of skeletal muscle homeostasis and provide compelling evidence that SIDT2 deficiency produces a muscular pathology with striking similarities to Danon disease, including accumulation of autophagic vacuoles with sarcolemmal features, selective atrophy of fast twitch fibers, and overlapping transcriptional alterations. Beyond confirming a role of SIDT2 in autophagic function, our data reveal that SIDT2 deficiency is associated with activation of type I interferon signaling and increased apoptosis, highlighting additional pathogenic mechanisms that may contribute to muscle degeneration. Together, these findings strengthen the functional link between SIDT2 and LAMP2 and support the notion that disruption of RNAutophagy-associated pathways can drive skeletal muscle pathology. Elucidating the molecular interactions between SIDT2, autophagy, and inflammatory signaling may not only advance our understanding of muscle biology but also uncover novel therapeutic targets for Danon disease and related lysosomal disorders.

## Data Availability

RNaseq data is publicly available at Gene Expression Omnibus, accession number GSE330932.

## Appendix A

**Table A1:** Comparison of Genes identified in the hearts of danon disease patients and differentially expressed genes in Sidt2 knockout mice muscle.

Gene	Common_name	Control_mean	Danon_mean	Fold_ change	*p* Value	FDR	Sig_ Mouse_ Sidt2KOvsWT
ENSG00000137168	PPIL1	52.05	19.81	−2.63	0.0000	0.0015	
ENSG00000005893	LAMP2	30.72	0.85	−36.14	0.0000	0.0095	Yes
ENSG00000101197	BIRC7	0.18	7.57	42.06	0.0000	0.0106	
ENSG00000100418	PPPDE2	18.61	12.28	−1.52	0.0000	0.0188	
ENSG00000115163	CENPA	0.62	9.1	14.68	0.0000	0.0188	
ENSG00000099822	HCN2	4.71	22.61	4.8	0.0000	0.0188	
ENSG00000159685	CHCHD6	5.77	13.01	2.25	0.0001	0.0234	
ENSG00000188176	SMTNL2	11.6	1.74	−6.67	0.0001	0.0234	Yes
ENSG00000074219	TEAD2	33.33	18.04	−1.85	0.0001	0.0279	
ENSG00000136235	GPNMB	82.66	307.47	3.72	0.0001	0.0279	Yes
ENSG00000215039	AC005840.1	11.78	19.62	1.67	0.0001	0.0289	
ENSG00000187773	FAM69C	3.31	10.6	3.2	0.0001	0.0289	
ENSG00000232656	IDI2-AS1	6.71	27.8	4.14	0.0002	0.0324	
ENSG00000120833	SOCS2	9.75	20.25	2.08	0.0002	0.0324	Yes
ENSG00000133028	SCO1	16.75	22.46	1.34	0.0002	0.0324	
ENSG00000109501	WFS1	38.2	17.12	−2.23	0.0002	0.0350	Yes
ENSG00000145244	CORIN	23.38	1.97	−11.87	0.0003	0.0385	
ENSG00000178988	MRFAP1L1	32.89	43.2	1.31	0.0003	0.0385	
ENSG00000111452	GPR133	21.23	6.39	−3.32	0.0003	0.0385	
ENSG00000163050	ADCK3	150.46	59.69	−2.52	0.0003	0.0385	
ENSG00000134291	TMEM106C	25.52	36.05	1.41	0.0003	0.0385	
ENSG00000174996	KLC2	15.33	9.5	−1.61	0.0003	0.0385	
ENSG00000166483	WEE1	16.7	33.41	2	0.0003	0.0391	
ENSG00000105968	H2AFV	74.66	92.51	1.24	0.0004	0.0392	
ENSG00000205363	C15orf59	35.36	14.88	−2.38	0.0004	0.0392	
ENSG00000143353	LYPLAL1	28.56	62.07	2.17	0.0004	0.0392	
ENSG00000160310	PRMT2	30.3	54.9	1.81	0.0004	0.0392	Yes
ENSG00000160783	PMF1	54.8	42.95	−1.28	0.0004	0.0392	
ENSG00000137959	IFI44L	4.28	10.81	2.53	0.0004	0.0392	
ENSG00000173156	RHOD	38.24	17.38	−2.2	0.0005	0.0392	Yes
ENSG00000117592	PRDX6	244.51	117.5	−2.08	0.0005	0.0392	
ENSG00000137575	SDCBP	94.19	55.09	−1.71	0.0005	0.0392	
ENSG00000149557	FEZ1	20.66	64.54	3.12	0.0005	0.0392	
ENSG00000172348	RCAN2	104.92	64.19	−1.63	0.0005	0.0402	
ENSG00000161570	CCL5	1.38	8.89	6.44	0.0005	0.0402	
ENSG00000126773	C14orf135	6.75	16.32	2.42	0.0006	0.0402	Yes
ENSG00000050426	LETMD1	12.52	22.34	1.78	0.0006	0.0430	
ENSG00000111666	CHPT1	87.92	53.52	−1.64	0.0006	0.0430	
ENSG00000163875	MEAF6	20.23	29.7	1.47	0.0006	0.0430	
ENSG00000175197	DDIT3	18.17	37.23	2.05	0.0007	0.0430	Yes
ENSG00000096968	JAK2	3.79	10.42	2.75	0.0007	0.0443	Yes
ENSG00000138639	ARHGAP24	5.4	12.48	2.31	0.0007	0.0443	Yes
ENSG00000141448	GATA6	18.52	26.01	1.4	0.0007	0.0443	
ENSG00000115561	VPS24	50.62	68.38	1.35	0.0007	0.0443	
ENSG00000168135	KCNJ4	10.97	24.83	2.26	0.0008	0.0452	
ENSG00000172005	MAL	2.57	8.07	3.14	0.0008	0.0458	
ENSG00000116717	GADD45A	23.72	75.7	3.19	0.0008	0.0469	Yes
ENSG00000122203	KIAA1191	31.35	41.4	1.32	0.0009	0.0493	Yes
ENSG00000129473	BCL2L2	17.28	25	1.45	0.0009	0.0493	
ENSG00000162458	FBLIM1	31.76	21.35	−1.49	0.0010	0.0493	
ENSG00000130222	GADD45G	21.95	100.32	4.57	0.0010	0.0493	Yes
ENSG00000115290	GRB14	11.83	2.23	−5.3	0.0010	0.0493	
ENSG00000206418	RAB12	34.88	53.73	1.54	0.0010	0.0493	Yes
ENSG00000241135	RP11-6F2.6	87.68	23.89	−3.67	0.0010	0.0493	
ENSG00000198734	F5	5.94	0.31	−19.16	0.0010	0.0493	
ENSG00000171766	GATM	6.82	20.5	3.01	0.0011	0.0493	Yes

**Table A2:** List of top 20 most differentially expressed genes in Sidt2^−/−^ muscles.

Symbol	Description	logFC	adj. P. Val
Msln	mesothelin	6.76	0.0011
Tmprss13	transmembrane protease, serine 13	6.38	0.0001
Ccdc110	coiled-coil domain containing 110	6.36	0.0000
Clca4a	chloride channel accessory 4A	5.99	0.0013
Tmem8c	transmembrane protein 8C	5.92	0.0042
Mmp12	matrix metallopeptidase 12	5.82	0.0012
Gsta1	glutathione S-transferase, alpha 1 (Ya)	5.67	0.0233
Bhlha15	basic helix-loop-helix family, member a15	5.51	0.0012
Gm3776	predicted gene 3776	5.46	0.0183
Derl3	Der1-like domain family, member 3	5.34	0.0072
Sln	sarcolipin	5.27	0.0003
Krt18	keratin 18	5.17	0.0004
Fgf21	fibroblast growth factor 21	5.15	0.0025
lincMD1	Long noncoding RNA MD1	5.08	0.0272
Slc6a7	solute carrier family 6 (neurotransmitter transporter, L-proline), member 7	4.99	0.0084
Fyb	FYN binding protein	4.91	0.0014
Ighg2b	immunoglobulin heavy constant gamma 2B	4.91	0.0032
Ahnak2	AHNAK nucleoprotein 2	4.91	0.0002
Sox11	SRY (sex determining region Y)-box 11	4.86	0.0084
Gdf15	growth differentiation factor 15	4.86	0.0057
